# Kaposi Varicelliform Eruption in a Chronic Kidney Disease Individual Under Tacrolimus: A Case Report

**DOI:** 10.1155/2024/8373606

**Published:** 2024-10-30

**Authors:** Seema Sitaula, Suraj Shrestha, Elisha Poddar, Rabin Gosain

**Affiliations:** ^1^Department of Dermatology, Tribhuvan University Teaching Hospital, Kathmandu, Nepal; ^2^Maharajgunj Medical Campus, Institute of Medicine, Kathmandu, Nepal

**Keywords:** herpes simplex virus, kaposi varicelliform eruption, renal transplant, tacrolimus

## Abstract

**Background:** “Kaposi varicelliform eruption” (KVE), also known as “eczema herpeticum,” refers to a rare widespread skin infection. The primary causal agent is thought to be the herpes simplex virus (HSV). Though common in patients with underlying skin dermatosis, systemic immunosuppression can at times lead to KVE.

**Case Presentation:** A 27-years male, a renal transplant recipient, under systemic immunosuppressants, presented with lesions over the whole body for 2 weeks and fever for 10 days. Skin examination revealed multiple flaccid vesicles with hemorrhagic fluid over the face, trunk, and bilateral extremities. Multiple erythematous erosions over the chest and abdomen, multiple petechiae and purpura over bilateral legs, palms, soles, and abdomen, erosions over the hard palate along with thick crusts over the scalp. A tzanck smear showed plenty of acantholytic cells. With a diagnosis of KVE, he was managed with an injection of acyclovir that led to resolution of his symptoms.

**Conclusion:** KVE is a serious condition that may have fatal outcomes. Early diagnosis and appropriate treatment of patients at risk for viral complications are very important medical considerations.

## 1. Background

“Kaposi varicelliform eruption” (KVE), also known as “eczema herpeticum,” refers to a rare and possibly fatal condition that causes a widespread skin infection and typically manifests as localized vesicular eruptions in a patient with an underlying cutaneous disease. The primary causal agent is thought to be the herpes simplex virus (HSV) [1, 2]. The disease is most frequently encountered in children because it co-occurs with underlying skin diseases, primarily atopic dermatitis (AD) [3]. Disruption of the epidermal barrier is the risk factor for KVE that is most clearly defined; however, underlying immunosuppressive conditions or treatments may also be involved. Numerous case reports of KVE in people with multiple myeloma and cutaneous T-cell lymphoma have been published [4, 5].

Eczema herpeticum has also been linked to topical and systemic immunosuppression, specifically topical tacrolimus [6]. According to Thappa et al.'s study, 17 out of 20 KVE patients were using immunosuppressive medications (such as systemic corticosteroids, cyclophosphamide, azathioprine, and cyclosporine) to manage the severity of their primary dermatosis [7]. Sporadically, methotrexate therapy has been linked [8].

Here, we describe a 25-year-old patient under immunosuppressants for renal transplant who developed KVE.

## 2. Case Presentation

A 27-year-male presented with lesions over the whole body for 2 weeks and fever for 10 days. The patient was apparently well 2 weeks back when he first developed itchy raised lesions over his scalp. The lesions soon turned to crusts and started spreading gradually to his face, chest, abdomen, and bilateral extremities. Some of his lesions turned into fluid-filled lesions which ruptured on their own and were nonitchy. Meanwhile, flat dusky-red lesions started appearing over his bilateral palms and soles. He also complains of painful lesions over his palate for 10 days which hindered his feeding. He gives a history of fever for 2 weeks. He also gives a history of backache and bilateral knee pain for 1 month. There is no history of hematuria, pain abdomen, photosensitivity, black stool, or use of any new drug prior to the appearance of lesions.

He is a known case of chronic kidney disease and underwent renal transplantation 4 years back and is under immunosuppressants (tacrolimus and corticosteroids). He also gives a history of hepatitis C 5 years back for which he took oral medications. There was no history of AD or previous similar skin lesions.

**Figure 1:**
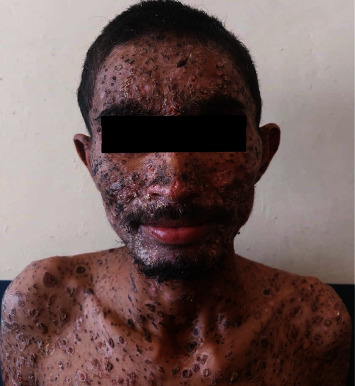
Multiple hyperpigmented crusted papules with scabs scattered all over the facial region involving the scalp.

**Figure 2:**
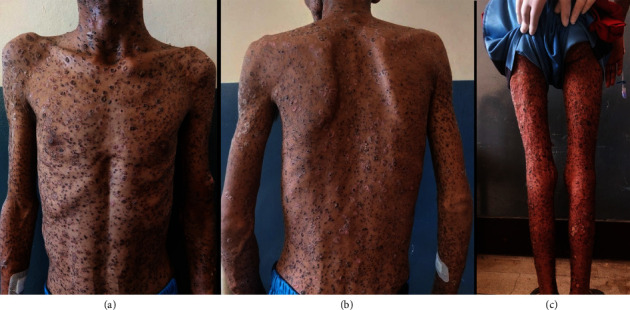
Multiple hyperpigmented crusted papules with scabs scattered all over the body.

On examinations, he had stable vitals. Skin examination revealed multiple flaccid vesicles with hemorrhagic fluid over the face, trunk, and bilateral extremities. Nikolsky sign was negative. Multiple erythematous erosions over the chest and abdomen; multiple petechiae and purpura over bilateral legs, palms, soles, and abdomen; erosions over the hard palate along with thick crusts over the scalp (Figures 1 and 2) were observed. An ophthalmological consultation was performed without showing any ocular complications.

**Figure 3:**
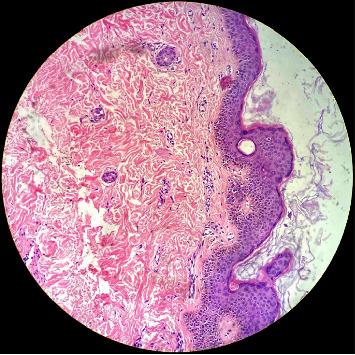
Histopathological examination of skin lesion shows intact epidermis with no vacuolar degeneration and minimal perivascular lymphocytic infiltration in the superficial dermis.

Laboratory investigations were unrevealing (Table 1). His serology was nonreactive. With a provisional diagnosis of KVE, a tzanck smear was done which shows plenty of acantholytic cells, neutrophils, foamy macrophages, and a few degenerated cells. Biopsy from the skin lesion showed epidermis with intact basal layer with no vacuolar degeneration and superficial dermis showed minimal perivascular lymphocytic infiltrate (Figure 3). Therefore, we concluded that the patient had a KVE secondary to immunosuppressed state considering both clinical presentation and pathologic findings.

**Figure 4:**
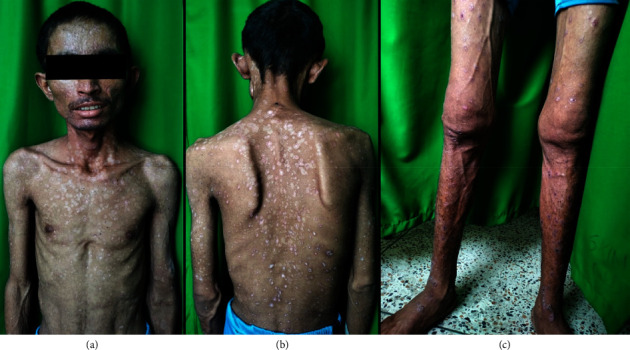
Multiple hypopigmented macules to papules noted all over the body after 14 days of admission (post–acyclovir therapy).

**Figure 5:**
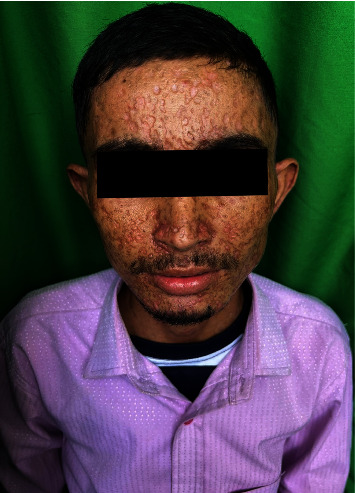
Multiple erythematous to hyperpigmented depressed plaques noted all over the face at 4 weeks of follow-up.

He was managed with an injection of acyclovir, IV fluids for adequate hydration, and other oral medications with the resolution of lesions with a week of antiviral therapy over 14 days duration. He was advised to follow-up with a nephrologist with the serum tacrolimus level. Four weeks after discharge, his lesions have healed well (Figures 4 and 5).

## 3. Discussion and Conclusions

KVE is an uncommon and potentially lethal viral illness that is primarily brought on by HSV reactivation, in addition to Coxsackie A 16 and varicella zoster virus [9].

An increased risk of developing KVE has also been linked to decreased production of certain cytokines, such as interferon-*γ* that may influence the formation of KVE and increase viral replication in the skin [8]. In this setting, cytokine production changes caused by immunosuppressive therapy can promote KVE development in people with skin problems. This may be the case with methotrexate, which has shown to lower levels of interferon [10]. In addition, our patient was on immunosuppressants as a renal transplant recipient which might have predisposed him for KVE development with mechanisms as described earlier. In fact, systemic immunosuppressive medication is used more frequently in adults, increasing the risk of KVE in individuals who are susceptible to it. It is thought that it results from cellular and humoral immune failure [11]. However, in patients who have previously had HS infection, the immunosuppressive impact of tacrolimus is a sensitive factor to trigger an extreme HS eruption (EEHS). Disseminated lesions may appear, making KVE difficult to distinguish from EEHS [12].

On skin afflicted by pre-existing dermatoses, KVE typically starts as an abrupt eruption of painful, edematous clusters of umbilicated vesiculopustules and may be accompanied by a flu-like symptoms. Vesiculopustules develop into throbbing, crusty, and punched-out erosions that are painful and hemorrhagic. These erosions combine to create denuded areas that are vulnerable to subsequent bacterial colonization [13]. With a sensitivity of 40%–80% and a specificity of up to 100% for the herpes virus, tzanck enables a quick diagnostic approach [14]. With a typical skin rash and acantholytic cells in tzanck smear, a diagnosis of KVE was made in our case.

Since KVE has the potential to be fatal, treatment must be started immediately. Antiviral therapy works well to lower morbidity and avoid problems including rhabdomyolysis, bacterial sepsis, and renal failure [15]. Since they prevent viral DNA replication, nucleoside analogs are the antiviral medications that are most frequently utilized. A nucleoside analog antiviral, such acyclovir, valacyclovir, or famciclovir, is the basis of treatment [16]. In newborns, immunosuppressed patients, and those with serious or systemic problems, intravenous delivery should be taken into consideration [17]. Unless there is subsequent bacterial infection, antibiotics are not recommended [18]. The most researched and frequently used medication for KVE is acyclovir. For the control of illness, high-dose intravenous acyclovir is frequently required. The skin lesions disappear for the majority of individuals over a few days. Prophylactic treatment with systemic antibiotics is recommended to prevent secondary bacterial infection [19]. Our patient was started on acyclovir along with other supportive therapy that led to resolution of symptoms within 10–14 days of initiation of medications.

KVE is a serious condition that may have fatal outcomes. Crucial medical considerations include prompt identification and treating patients at risk for viral problems appropriately.

## Figures and Tables

**Table 1:** Various laboratory parameters at the time of admission.

Laboratory parameters	At admission
Complete blood count	
1. Hemoglobin	12.1
2. Total leukocyte count	15,000
3. Platelets	136,000
Renal function test	
1. Urea/creatinine	14.6/98
2. Na/K	131/3.8
PT/INR	16/1.3
Liver function test	
1. TB/DB	19/5
2. AST/ALT	114/78
3. ALP	123
Serology	Non-reactive
Urine RE/ME	Normal
24 h urinary protein	0.42 g

## Data Availability

All the necessary data and materials are provided within the manuscript.

## Nomenclature

AD: Atopic dermatitis
HSV: Herpes simplex virus
KVE: Kaposi varicelliform eruption
